# Reading Strategies for Children with Developmental Language Disorder

**DOI:** 10.3390/children9111694

**Published:** 2022-11-04

**Authors:** Gema De las Heras, Teresa Simón, Ana B. Domínguez, Virginia González

**Affiliations:** 1 Faculty of Health Sciences, University of Castilla La Mancha, 45600 Talavera de la Reina, Spain; 2 Faculty of Psychology, Complutense University of Madrid, 28223 Madrid, Spain; 3 Faculty of Education, University of Salamanca, 37008 Salamanca, Spain

**Keywords:** developmental language disorder, reading, keyword strategy

## Abstract

Developmental language disorder (DLD) is considered a neurodevelopmental disorder that compromises language comprehension and/or expression and constitutes a risk factor for learning to read. The aim of the present study was to analyse the reading strategies used by students with DLD to read sentences. There is evidence in the literature that, when linguistic resources are insufficient, the keyword strategy (identifying some of the words in the sentence with their own semantic content, with barely no processing of the function words) is used to read sentences. A total of 31 primary and secondary school students diagnosed with DLD were evaluated using the PEALE battery. The results reveal that students with DLD present heterogeneous profiles that are below the established reading level for their age. In addition, children with DLD and better reading skills use the keyword strategy to read sentences. In conclusion, clinical and educational implications for reading intervention in individuals with DLD are discussed.

## 1. Introduction

Developmental language disorder is considered a severe and persistent disorder, not associated with a biomedical condition, that heterogeneously compromises oral language acquisition and development and affects social development and learning throughout life [1]. In this regard, the possible impacts of long-term developmental language disorder (DLD) on communication and learning have guided the focus of research to establish an “academic prognosis”, that is, how oral language difficulties influence the literacy development process and/or in determining which difficulties in this process predict future reading difficulties [2,3]. 

Research on DLD has shown that oral language difficulties and reading difficulties are related [4]. Specifically, relationships have been established between the development of certain areas of oral language, such as lexis, morphosyntax and phonology, and reading comprehension [5,6]. In fact, children with oral language difficulties present a higher risk of reading problems compared to a typically developing group [7]. 

In this sense, the simple view of reading (SVR) [8] explains the convergence/divergence of oral language and word recognition skills in those learning to read. This was extensively documented in the National Reading Panel report [9] and recently revised by its authors [10,11]. The SVR argues that reading comprehension is the product of two factors: word recognition and language comprehension. Nonetheless, in the original proposal of the SVR, the ability to recognize printed words was referred to as decoding but is now better expressed by the broader term of word recognition, which includes both more indirect recognition through grapheme-phoneme correspondence and more direct recognition through orthographic mapping [10]. Its constructs can be defined as follows [10]:

- *Reading comprehension is the ability to extract and construct literal and inferred meaning from linguistic discourse represented in print*.
- *Language comprehension is the ability to extract and construct literal and inferred meaning from linguistic discourse represented in speech*.
- *Word recognition is the ability to recognize printed words accurately and quickly to efficiently gain access to the appropriate word meanings contained in the internal mental lexicon*.

In other words, the SVR fundamentally includes language skills that are common to the comprehension of oral and written text, that is, the reader’s lexical competences (or knowledge of word sense), as well as the processes of morphosyntactic analysis and semantic integration (see [12] for a review). Drawing on this model, prior studies on the reading skills of students with DLD have yielded heterogeneous findings that include students with DLD without reading difficulties, with deficits in reading comprehension, and/or students that present a mixed profile of difficulties [13,14].

It is evident that, in order to understand a text correctly, a reader must know most of the words of which it is made up, as well as the syntactic structures it uses. In Spanish, there are studies that have shown evidence of the deficiencies that students with DLD present in oral expression of function words, omitting, in particular, articles and prepositions [15]. In relation to pronouns, various studies conclude that children with DLD perform worse in the use of clitic pronouns (e.g., [16]). Furthermore, it has been reported that the presence of a morphosyntactic difficulty at the oral level may increase the likelihood that morphosyntactic difficulties manifest in other learning modalities, such as reading and writing, which in turn may increase the risk of school failure [17]. Consequently, we can hypothesize that the difficulties in function and syntactic word processing presented by the group of students with DLD may explain their difficulties in learning to read.

The question thus arises on how students with DLD read sentences if their syntactic skills are impaired. What strategy do they use in reading? Similarly, why do they use this strategy and not another? The hypothesis explored in this study is that a reader with DLD will tend to adopt a particular strategy for sentence reading, which Domínguez and her collaborators call keyword strategy (KWS) [18]. This strategy consists of selecting words with their own semantic content (nouns, verbs, and adjectives) and inferring the meaning of the text without processing the function words (prepositions and conjunctions). For example, in a sentence such as *Luis lee muy bien* (Luis reads very well), when using the KWS, the words *Luis*, *lee,* and *bien* are likely to be considered. To demonstrate the use of this strategy, the battery of the Test for Analytical Evaluation of Reading (PEALE [19], in its Spanish acronym) was developed, in which comparing performance on two formally identical reading tasks (a basic reading efficacy test and the keyword strategy detection task, task descriptions below) serves as the empirical evidence of the use of this strategy. The tasks presented to the participants consist of a series of written sentences in which a word is missing, and where they are required to choose one of four options to complete it correctly. The result is compared between the basic reading efficiency test (TECLE, in its Spanish acronym), in which the options are orthographically similar, and the keyword strategy detection task is considered, where the distractors are semantically compatible with the keywords in the sentence. If we take the example above, the sentence *Luis lee muy bien* (Luis reads very well) is presented, and four possible answers are provided: the correct one and three distractors that are semantically compatible with the meaning of the sentence, *libros biblioteca* and *periódico* (books, library and newspaper). The underlying logic is that a reader using the KWS will have greater difficulty in the task with distractors compatible with the meaning derived from the keywords than in the basic reading task [20].

In this regard, studies have shown that readers with poor reading skills rely on semantic context to compensate for their difficulties in identifying written words, which could lead them to adopt the KWS [21].

In light of the abovementioned studies, it is of interest to investigate the possibility that children with DLD use the KWS. Furthermore, whatever the findings, a more detailed exploration of the reading strategies used by children with DLD may help educational intervention with these students.

Thus, the first aim of this study is to establish the type of reading strategies used by students with DLD to reach their reading level. More specifically, the intention is to determine whether the keyword strategy (KWS) is used by students with DLD to extract the information contained in a sentence, given their lack of linguistic resources. The second aim is to analyse the relationship between the use of the KWS and syntactic skills in reading (more precisely, the ability to deal with function words).

## 2. Materials and Methods

### 2.1. Participants

Thirty-one students with a prior diagnosis of DLD, aged over 6 years (mean = 9.75 years; SD = 2.68; range: 6.3 to 15.4 years) participated in this study. At the time of the assessment, all the children (except two) were engaged in out-of-school speech therapy. They were all enrolled at schools in the region of Castilla—La Mancha (Spain). For the diagnosis of DLD, the criteria established by [22] were followed, including evaluation with the CELF-4 (Clinical Evaluation of Language Fundamentals; [23]). The comparison group comprised 797 students with typical reading development (395 females and 402 males), with a mean age of 8.80 years (SD = 0.06; range 6.0 to 12.1), and with no type of language difficulty or physical, sensory, or cognitive disability. They were recruited from 11 different cities in Spain, and all of them attended school grades corresponding to their chronological age. All children of the sample were monolingual Spanish speakers.

The initial research interest in establishing a reading profile of students with DLD gave rise to the study of the heterogeneity of the intragroup reading profile [24]. Consequently, as criteria for the creation of subgroups, authors such as [6,7] establish the presence or absence of reading difficulties, including dyslexia/DLD comorbidity [2]. Specifically, Catts [24] in their study compared the language profiles of children with a history of language deficits, thus establishing two reading groups based on their reading comprehension performance and word recognition composite scores, with a cut-off point set at the 25th percentile.

In the present research, to examine the variation in reading levels within the group of students with DLD, we established two subgroups following the criterion of reading performance/delay [25]. To establish the two subgroups, we used the procedure followed in previous studies [20,26] based on the TECLE Reading Test [27] (described in the section on instruments). For this purpose, as a reference, we used the cut-off score on the TECLE test below which a severe reading delay is considered. This cut-off score is calculated as follows: mean TECLE score (for each school grade)—1.5 SD × SD of the age-appropriate scores provided in this test. The 31 students with a DLD diagnosis were divided into two subgroups: the first subgroup comprised 18 students with DLD and mild reading delay (MRD), aged between 6.33 and 11.42 years (M = 8.26; SD = 0.38), while the second was made up of 13 students with DLD and severe reading delay (SRD), aged between 7.75 and 15.42 years (M = 11.72, SD = 0.71).

### 2.2. Instruments

The main aim of the present research was to evaluate the reading strategies of students with DLD use to read sentences. To this end, we administered three reading tasks taken from previous works by [19]. Specifically, we administered the TECLE reading efficiency test [27] and two tests from the PEALE battery (the keyword strategy detection test (KWS) and the test of syntactic ability test (SNT)). These tests can be downloaded free of charge at https://complydis.usal.es/ (accessed on 21 august 2022)

For each of these tests, the percentage of correct answers was calculated for each participant, adjusted to eliminate the effect of random correct responses, using the following formula—the number of correct answers minus the number of errors, divided by three (A- (E/3)).

#### 2.2.1. Reading Level Test

To assess the children’s reading level, we used the Collective Test of Reading Efficiency (TECLE) [27]. This consists of 64 sentences with a missing word, which participants are required to complete using one of the multiple-choice possibilities. For example, in the sentence *El caballo tenía la pata*… (The horse’s leg was …), the participant is given four options, from which they are asked to choose the one that correctly completes the sentence: the correct word (*rota*-broken), two pseudo-words that are orthographically and phonologically similar to the correct answer (*rola* and *roka*), and a word that is semantically inconsistent with the sentence (*ropa*-clothes).

Participants are given 5 min to complete as many sentences as possible. Prior to administering the test, the instructions are explained, and three example sentences are completed. The sentences in the test appear in increasing order of difficulty, according to variables such as length and syntactic complexity, as well as the frequency of the words of which they are composed. The reliability measured using Cronbach’s alpha and the two-halves test is high (*α* = 0.966 and *r* = 0.986, respectively) [18].

#### 2.2.2. Keyword Strategy Detection Test (KWS)

This test assesses the use of the keyword strategy (KWS). Similarly to TECLE, it is composed of 64 incomplete sentences. In this case, the distractors are semantically compatible with the sentence. Returning to the example used in the introduction, *Luis lee muy*… (Luis reads very …), the correct answer is *bien* (well), and the three distractors are *libros, biblioteca,* and *periódico* (book, library, and newspaper), all of which are compatible with the overall meaning generated with the probable keywords (*Luis*, *lee*). A reader using the keyword strategy might choose one of the options—*libros*, *biblioteca*, or *periódico*—as a correct word to complete the sentence. Only an exhaustive analysis of the function words allows the reader to exclude those words and use *bien* as a grammatically compatible solution. The participants are given five minutes to complete the sentences once they have completed the three trial examples. As was the case in the Reading Level Test, the difficulty of the task increased from the first sentence onwards. Reliability for this test, measured using Cronbach’s alpha and the two-halves test, is also high (0.980 and 0.992) [18].

The proposed criterion for establishing the extent to which participants use the KWS is to compare the delay on the basic reading test (TECLE) with the delay on the KWS task. The term “delay” is operationalized by taking the difference between the age at which each participant achieved a specific score and the age at which the control group (typical readers) achieved the same score (for a detailed explanation with examples, see the Results section). Greater difficulties on the KWS task, compared to performance on the TECLE test, are evidence of the use of the KWS, as it shows a tendency to pay less attention to function words.

#### 2.2.3. Syntactic Ability Test (SNT)

The purpose of this test is to assess function word processing in sentence reading. To this end, participants are required to complete a series of sentences that are missing a function word. The structure is the same as in the previous tests, that is, 64 sentences and four response options, but, in this case, all the distractors are function words (conjunctions, prepositions and adverbs). For example, in the sentence *Mañana llega… Madrid* (*Tomorrow she’s arriving … Madrid*), the participant is required to choose between the correct option *de* (from), which is the only one that correctly introduces the complement Madrid, and the three distractors, *con, para,* or *entre* (with, for, or between), which form grammatically incorrect sentences.

According to previous studies [18,20], choosing one of the distractors reveals difficulties at the syntactic level. In this sense, and in contrast to the previous tests, the reading dimension of the task is reduced by using familiar, frequently used words, short sentences (4 to 6 words across the complete test), and simple syntactic structures (mainly subject, verb, and a complement) to prevent participants from drawing on the context when choosing between the options presented.

The procedure followed to apply this test was the same as that used with the TECLE and the KWS. Reliability for this test, measured using Cronbach’s alpha and the two-halves test, is also high (*α* = 0.979 y *r* = 0.991) [18].

### 2.3. Procedure

Prior to the study, all the families signed an informed consent form for participation and confidentiality. The students with typical reading development were evaluated at their school, collectively. Meanwhile, the children with DLD were assessed individually. Their assessment tests were administered in speech therapy clinics and associations in Castilla—La Mancha and were divided into two sessions, one devoted to assessing reading performance (TECLE) and detecting the use of the keyword strategy (KWS), and the other dedicated to evaluating syntactic ability. Prior to the start of each test, and after having explained what it consisted of, three examples were given to ensure the task was understood.

### 2.4. Data Analysis

To analyse the data, we used the same method as that followed in previous studies with deaf students [18] and with students with dyslexia [20]. This was because, in order to compare the results of two groups in a task considering their mean score, both groups must necessarily be matched to other variables that affect this score, such as age or reading level. In this study, we opted to use a method based on linear regression analysis, in which the performance of each participant (dependent variable expressed as the score obtained in each test according to its assessment criteria) is evaluated in relation to their chronological age in months (independent variable). This method allows individual delays for each task to be calculated by comparing a participant’s score with the whole comparison group represented by the group regression equation, instead of considering a few participants who have approximately the same age or the same reading level as the participant being evaluated. In the results section, we will explain and report the slopes, intercepts, and Coefficient of Determination of the regression equations.

To make between group comparisons, the assumptions of normality were tested using the Shapiro-Wilk test and the equality of variances (Levene’s test). A single-factor parametric analysis of covariance (ANCOVA), with chronological age as a covariate, was also applied. The level of significance was set at 0.05. All data analyses were conducted with the SPSS program, version 25.0.

## 3. Results

We present the results task by task, following the same system. Linear regression equations were calculated by group and task, considering the percentage of correct answers in each task, in relation to the participant’s age. Table 1 shows the parameters of the regression equations by task and group according to age where: b is the slope of the line, explaining the variation of performance in each of the tests according to participant age, a is the value of the equation when age takes a value = 0, and *R*^2^ is the coefficient of determination used to predict future results or test a hypothesis. The coefficient determines the quality of the model to replicate the results and the proportion of variation in the results that can be explained by the model. In simple regression models, this value is the square of the Pearson’s correlation coefficient. 

The individual delays in each task were calculated by taking the difference between the age at which each participant achieved a specific score and the age at which the control group (typical readers) achieved the same score. For example, in the TECLE test (y = 0.815X − 45.002, for the sample considered in this study), the reading delay (RD) score was obtained, which is expressed by the difference between the score obtained by the participant in the reading level test, TECLE, and that of the typically developing group of the same age: RD = TECLE − (0.815 × age − 45.002). A negative value shows a RD with respect to children of the same age. By definition, the RD in the typically developing group would be 0.

The same procedure was used to measure delays in the KWS (semantic delay) and STX (syntactic delay) tasks. The mean delays in years per group in each of the tests can be seen in Table 2.

### 3.1. Assessment of the Keywords Strategy

To determine whether the students with DLD with both MLD and SLD used the keyword strategy (KWS) to read sentences, the scores on the KWS test were used and the corresponding delays were calculated, as explained in the previous section. To analyse the use of this strategy, the delays on the TECLE test were compared to those obtained on the KWS task; greater delays in the latter test than in the TECLE reading test are indicative of the use of the KWS. Table 2 shows the mean delays in the TECLE and KWS tasks by age. It can be seen that the group with DLD-MRD has more difficulties on the KWS task (−1.45) than on the TECLE (−0.92). While in the DLD-SRD group there is very little difference between the two tasks (−4.87 and −5.21 respectively).

To address the aim of analysing the ability to deal with function words in children with DLD we performed an analysis of covariance taking SNT delay as the dependent variable, group as a fixed factor, and age as a covariate The results of the ANCOVA showed a significant effect of group (*F*(2, 824) = 83.09, *p* =0.001, *η* ^2^*p* = 0.17) and significant differences between all groups (*p* < 0.001). These data underline that the use of the keyword strategy may mean that readers who use it have to apply different resources and linguistic knowledge than students who do not use this strategy or use it to a lesser extent.

To examine statistically the tendency to use the KWS, we have adopted the most direct index reflecting this tendency (I-KWS), which is the difference between RD and KWS-D (I-KWS = RD − KWS-D). The null hypothesis was that this difference should be zero, as is the case in typically developing group, if DLD and TD participants adopted the KWS to the same degree. Positive values corresponded to greater delays in the KWS test than in the TECLE test due to the adoption of the KWS. The ANOVA on I-KWS as a dependent variable per group using age as covariate showed significant effect on the group (*F*(2824) = 3.24, *p* = 0.036, *η* ^2^*p* = 0.008). The covariate age was not significant (*p* = 0.987). The group with DLD-MRD had more difficulties on the KWS test than on the TECLE test (mean I-KWS = 0.53). In the DLD-SRD, this index was negative and showed an opposite pattern—better performance on the KWS test than on the TECLE test (mean I-KWS = −0.35).

**Figure 1:**
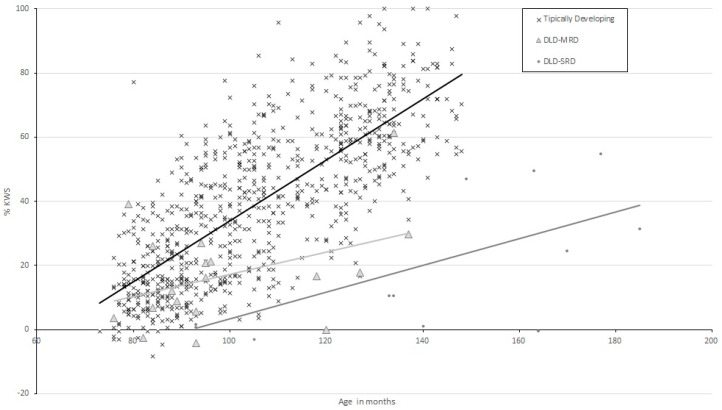
Scores for performance on the KWS test (in %) according to age in months by group (typically developing, DLD-MRD, and DLD-SRD).

Figure 1 shows the performance on the KWS test according to age for these three groups. Visual observation of Figure 1 suggests that typically developing participants’ performance on this task is better in older participants, as is the case for the DLD groups, but with a slighter/more subtle improvement as participant age increases, if we consider the slopes of the regression lines (see Table 1).

### 3.2. Assessment of Syntactic Skills

The second aim of this work was to determine how children with DLD-MRD make use of the KWS. The hypothesis was that the use of the KWS stems from difficulties in processing function words in sentence reading. Consequently, the students were expected to find the SNT task more complex than the TECLE test. Table 2 shows the mean syntactic delays (STX-D) by group, calculated in the same way as the RDs. As in the previous tests, even the group of participants with DLD-SRD group of participants have high STX-Ds when compared to the reading levels obtained. Figure 1 shows the mean delays in TECLE and SNT according to age. It can be seen that the students with DLD-MRD performed worse on the SNT (−1.44) compared to the TECLE (−0.92). The DLD-SRD group showed a delay of −5.21 years in the latter test and of −4.71 years in the SNT. To address the aim of analysing the ability to deal with function words in children with DLD we performed an analysis of covariance taking SNT delay as the dependent variable, group as a fixed factor, and age as a covariate. The ANCOVA performed showed a significant group effect (*F*(2, 824) = 87.21, *p* < 0.001, *η* ^2^*p* = 0.18) and significant differences between all the groups (*p* < 0.001). These results confirm the hypothesis that one of the possible explanations for the use of the KWS in students with DLD may be their explicit difficulties in dealing with function words.

If we look at the parameters of the regression equations (Table 1), the trend of progress in syntactic skills in the three groups increases with student age. The value is higher, however, for the typically developing group of students (0.878) compared to their counterparts with DLD-MRD (0.483) and DLD-SRD (0.444). These scores show a significant difference in progress with respect to the typically developing group.

## 4. Discussion

The general aim of this work was to analyse the reading strategies used by students with DLD to read sentences. Two subgroups were formed following the criterion of reading performance [25] and considering that the children with DLD present heterogeneous profiles of reading difficulties (from low or non-functional performance to almost normal levels), similar to those found in previous studies [13,28,29,30]. On the one hand, a DLD-SRD subgroup with a marked reading delay (−5.21 years) compared to the typically developing group and, on the other, a DLD-MRD subgroup with a lower reading delay (−0.92 years).

An interesting notion is that all the groups analysed increase in reading performance with age.

However, the improvement in reading tasks expected in older children in the DLD-MRD and DLD-SRD subgroups is three times less than that of the typically developing group. It has also been shown that difficulties in reading performance do not mitigate over time [31], but persist, and the gap between these children and their peers increases [32]. It has been found that even students with DLD who had a good start in learning to read showed slower development in decoding skills [33]. Consequently, DLD students with reading difficulties can be considered to improve so slowly that, with age, the gap between them and typically developing children widens. Moreover, the older the students are, the lower the effectiveness of reading intervention [34].

Furthermore, the slowness of the improvement in reading skills in people with DLD underscores the importance of preventing reading difficulties and providing early educational/clinical care [35]. It is therefore essential to adopt a proactive model in response to reading difficulties and to prevent such deficits from impacting subsequent learning [36]. Indeed, the response to intervention (RtI) model, the strength of which lies precisely in focusing on prevention, has yielded promising results in language intervention in individuals with DLD [37,38] and in reading in the dyslexia population [39].

The first aim was to examine the reading strategies students with DLD use to read sentences, particularly whether students with DLD use the keyword strategy to read sentences. This strategy consists of identifying frequently used words with full semantic content to build a global meaning, with minimal processing of function words [18].

Our analysis of the reading strategies used by students with DLD reveals that the DLD-MRD subgroup makes use of the keyword strategy. Their use of the KWS is evidenced by the fact that their delay in the task with semantic distractors (KWS: −1.45 yrs) was greater than that in the basic reading task (TECLE: −0.92 yrs). This suggests that, to read sentences, they rely more on semantic strategies and less on processing function words, in particular. The findings of our study corroborate, on the one hand, the idea that students with DLD have specific difficulties with the processing of function words and, on the other hand, that the group with DLD-MRD uses semantic strategies to a greater extent than the typically developing group because their syntactic skills are poorer. All in all, the results obtained suggest that the analysis of keyword strategy use may be an important element missing in studies on reading in populations with DLD.

In contrast, the DLD-SRD subgroup showed no additional delay in the KWS task (DEPC) in relation to the basic reading task, so we cannot categorically claim they use the KWS. Nonetheless, the results indicate significant and constant delays in all the areas evaluated, giving rise to a profile of persistent and generalized reading difficulties [40].

The results might suggest that treating language difficulties in children with DLD does not appear to reduce the risk of reading problems [40]. However, we should be cautious in this regard since we have taken into account that all the students received out-of-school speech therapy without assessing aspects such as the type or duration of the treatment of language difficulties. As a possible future proposal, it would also be interesting to investigate to what extent the schooling process influences reading improvement in students with DLD.

The second aim of this study was to examine why learners with DLD use the KWS. To this end, we analysed syntactic competence, since, as discussed in the introduction, explicit difficulties in the handling of function words support the use of KWS in other groups [18]. In the present study, the DLD-MRD group presents a significant delay in the SNT test compared to the typically developing group. This was to be expected given there is evidence of syntactic deficits in DLD, which has even been considered a diagnostic indicator of DLD [41,42]. The data obtained in our research appear to support the hypothesis that one of the possible explanations for the use of the KWS in students with DLD-MRD lies in their deficits in syntactic abilities [20]. Furthermore, comparing the delays found in the SNT task, it can be seen that the DLD-MRD group shows a delay of approximately 1.5 years compared to the typically developing group. This might be because, in the tasks that assess non-specific reading skills, i.e., syntactic and lexical skills, the students with DLD performed worse, since they start learning to read with fewer linguistic resources and progress much more slowly than typically developing students [20].

The data presented in this study, overall, advance the knowledge of the reading skills of children with DLD across the school years. However, the heterogeneity of the group of students with DLD, as well as the size of the sample, limit the extrapolation of our results.

Additionally, there exist many comorbidities that are not assessed in this study, and which, as regards reading, may be of great importance.

However, the heterogeneity of the profiles and difficulties of students with DLD in reading require an analysis of the extent to which word identification processes and language skills, particularly vocabulary and syntax, are affected. In this sense, the analytical assessment of reading provides a means to delve deeper into the nature of reading delay, thus formulating hypotheses about the linguistic and cognitive resources used by students to reach a certain reading level. Specifically, the PEALE battery [19] has proven to be a valid and effective tool for assessing the reading strategies of students with DLD, yielding the necessary information to design practices in teaching reading that are adapted to the needs of the individual child.

The findings on reading skills presented in this study have important implications for educational practice since reading is one of the fundamental processes underlying a large number of school activities, as well as many of those of daily life. This study provides evidence for the role of the keyword strategy in the sentence reading of students with DLD. It would be useful to conduct further studies on KWS use in adults with DLD to determine whether, as in the deaf population, it is a strategy that is maintained across the lifespan, and which also provides evidence of the need for explicit and systematic syntactic intervention.

Nevertheless, the results of the subgroup with DLD and poorer reading performance underline a challenge in educational and clinical practice. That is, on the one hand, we should question the efficacy of the support offered to students with DLD across the school years and beyond, and on the other, there is a need to provide evidence-based reading strategies that facilitate such students’ learning, enhance their motivation to continue with school, and maximize their opportunities for personal, social, and occupational development.

In short, it is necessary to conduct longitudinal research and make proposals for explicit intervention in reading to establish relationships that explain the heterogeneity of results and, thus, design an intervention appropriate to the strengths and difficulties experienced by each individual schoolchild at different points of their life. It is also critical that experimental designs are used to test the efficacy and effectiveness of such interventions.

## Figures and Tables

**Table 1:** Slopes (b), intercepts (a), and coefficient of determination (*R*^2^) of the regression equations calculated on the percentage of correct answers on the TECLE, KWS, and SNT tasks, according to age, for the different groups (typically developing, DLD-MRD, and DLD-SRD).

TECLE				KWS			SNT		
	a	b	$R^{2}$	a	b	$R^{2}$	a	b	$R^{2}$
Typically developing	−45.002	0.815	0.605 ***	−61.021	0.949	0.579 ***	−54.842	0.878	0.604 ***
DLD-MRD	−8.149	0.357	0.381 **	−17.185	0.345	0.167	−30.460	0.483	0.375 **
DLD-SRD	−24.905	0.329	0.349 *	−38.318	0.416	0.367 *	−40.487	0.444	0.382 *

Note: DLD-MRD = Developmental language disorder with mild reading delay; DLD-SRD: Developmental language disorder with severe reading delay, *** *p* ≤ 0.001; ** *p* ≤ 0.01; * *p* ≤ 0.05.

**Table 2:** Mean reading, semantic, and syntactic delays by group (typically developing, DLD-MRD, and DLD-SRD) in years.

	Reading Delay (TECLE)	Semantic Delay (KWS)	Syntactic Delay (STX)
Typically developing	0.00	0.00	0.00
DLD-MRD	−0.92	−1.45	−1.44
DLD-SRD	−5.21	−4.87	−4.71

## Data Availability

Not applicable.
